# Influence of Tendon Location on the Clinical Response to Platelet-Rich Plasma: A Prospective Cohort Study of Rotator Cuff, Achilles and Patellar Tendinopathies

**DOI:** 10.3390/jcm15052005

**Published:** 2026-03-05

**Authors:** Mikel Sánchez, David Santos-Hernández, Cristina Jorquera, Jaime Oraa, Renato Andrade, João Espregueira-Mendes, Fernando Yangüela, Sergio González, Jorge Guadilla, Diego Delgado

**Affiliations:** 1 Arthroscopic Surgery Unit, MiKS Hospital, 01010 Vitoria-Gasteiz, Spain; mikel.sanchez@hospitalmiks.com (M.S.); david.santos@hospitalmiks.com (D.S.-H.); jaime.oraa@hospitalmiks.com (J.O.); fyanguela@rmagnetica.com (F.Y.); sergio.gonzalez@hospitalmiks.com (S.G.); jorge.guadilla@hospitalmiks.com (J.G.); 2 Advanced Biological Therapy Unit, MiKS Hospital, 01010 Vitoria-Gasteiz, Spain; cristina.jorquera@hospitalmiks.com; 3 Clínica Espregueira—FIFA Medical Centre of Excellence, 4350-415 Porto, Portugal; randrade@espregueira.com (R.A.); jem@espregueira.com (J.E.-M.); 4 Dom Henrique Research Centre, 4350-415 Porto, Portugal; 5 Porto Biomechanics Laboratory (LABIOMEP), Faculty of Sports, University of Porto, 4200-450 Porto, Portugal; 6 School of Medicine, University of Minho, 4710-057 Braga, Portugal; 7 ICVS/3B’s-PT Government Associate Laboratory, 4710-057 Braga, Portugal; 8 3B’s Research Group—Biomaterials, Biodegradables and Biomimetics, University of Minho, Headquarters of the European Institute of Excellence on Tissue Engineering and Regenerative Medicine, 4805-694 Barco, Portugal

**Keywords:** platelet-rich plasma, Achilles tendon, patellar tendon, rotator cuff, growth factors

## Abstract

**Background/Objectives**: Platelet-rich plasma (PRP) has become a therapeutic option for tendinopathies. Its clinical efficacy depends on several factors, including the target tendon. The aim of this study was to evaluate the PRP efficacy for tendinopathies in the rotator cuff (RC), Achilles tendon (AT), and patellar tendon (PT). **Methods**: We conducted a prospective cohort study including patients with RC, AT and PT tendinopathies. Each patient received three multitarget PRP (intratendinous and peritendinous) treatments at intervals of two weeks. Clinical outcomes were assessed at baseline and 6 months using tendon-specific scores (DASH for RC, VISA-A for AT and VISA-P for PT). Responders were identified based on the Minimal Clinically Important Improvement (MCII). Comparative statistical tests and multivariate regression were performed for the analysis. **Results**: A total of 49 patients were included (RC: 15, AT: 18, PT: 16). The number of responders at 6 months was 33 (67.4%), with 11 (73.3%) in the RC Group, 14 (75.0%) in the AT Group and 8 (50.0%) in the PT Group. The RC and AT patients experienced a significant improvement according to their scores (*p* < 0.001), which was not seen in the PT group (*p* = 0.065). The percentage of responders was higher in women (12/13, 92.3%) than men (21/36, 58.3%) (*p* = 0.025). **Conclusions**: Repeated intratendinous and peritendinous PRP injections in RC, AT, and PT tendinopathy improved joint-related function six months after treatment. This improvement was less pronounced in patients with PT and the proportion of responders was higher among women.

## 1. Introduction

The primary function of tendons is to transmit forces that enable joint movement. This mechanism subjects the tendons to constant mechanical stress and, consequently, increases the risk of overuse injuries [1]. Without an adequate recovery time, the tendon’s slow collagen regeneration can lead to degenerative changes that cause significant pain and impair overall function [2].

The pathophysiological mechanisms underlying tendinopathy are not yet fully understood, with no consensus on which approach most effectively improves patient outcomes. Therapeutic strategies vary depending on the severity of the condition, the intensity of pain, symptom duration, and patient adherence [3]. Traditionally, conservative treatment involves rest, local cold application, and therapeutic exercises, while surgical interventions are reserved for the most severe or refractory cases [3,4,5]. Eccentric as well as isometric exercises are considered the most effective therapeutic approach for most tendinopathies, particularly when implemented as intensive programs compared to other exercise modalities [6]. In the short term, corticosteroid use has demonstrated some efficacy, and its effects may be enhanced when combined with ultrasound-guided lavage. However, prolonged corticosteroid use is known to be ineffective and may even be detrimental due to its degenerative impact on the tendon [7].

In recent years, the use of Platelet-Rich Plasma (PRP) has gained popularity in the treatment of tendinopathies [8]. Its therapeutic effect is attributed to its platelet concentration and the subsequent release of growth factors and other bioactive proteins that play a crucial role in tendon tissue repair. PRP is a safe, autologous treatment derived from the patient’s own blood, which is easy to obtain and apply, and can be administered using minimally invasive techniques or as a biological augmentation during surgery [9].

Laboratory studies have demonstrated the beneficial effects of PRP in repairing tendon injuries. PRP stimulates the proliferation of the two primary cell types in tendons: tenocytes and tendon stem or progenitor cells [10,11]. PRP has also been shown to induce the differentiation of progenitor cells into tenocytes [12], as well as modulate inflammatory processes by stimulating the release growth factors with anti-inflammatory properties, such as hepatocyte growth factor (HGF), which is associated with a reduction in pro-inflammatory mediators such as COX-1, COX-2, and PGE_2_ [13].

Despite evidence from experimental studies, clinical research presents many contradictory results regarding the efficacy of treatments for pathologies affecting the Achilles tendon [14], rotator cuff [15] or patellar tendon [16]. One primary reason for this inconsistency is the lack of standardization in treatment PRP preparation protocols, with significant variability related to the PRP composition and administration [17], such as varying in leukocyte content, platelet concentration, as well as both form (intratendinous or peritendinous) and volume of administration [18]. Another important variable is the specific pathology and anatomical area being treated. The literature suggests that some tendons respond better to treatment than others [19]; however, due to variability in other factors, it remains difficult to draw definitive conclusions.

The aim of this study was to evaluate the efficacy of PRP in treating tendinopathies in the rotator cuff, Achilles tendon, and patellar tendon, and to analyze the differences between these groups. Our hypothesis was that, when applying the same type of PRP product and following the same administration protocol, the efficacy of PRP will vary depending on the target tendon.

## 2. Materials and Methods

### 2.1. Study Design

This prospective observational study included all consecutive patients meeting the inclusion criteria who were recruited between 2021 and 2024 at the same medical center. It was carried out in accordance with the international Declaration of Helsinki (Helsinki, Brazil; 2013), Good Clinical Practice and the STROBE statement. Ethical approval (protocol no: EPA2016067) was obtained from the Ethics Committee of the Basque Country (February 2017), and informed consent was obtained from patients. The study included patients with tendinopathy of the rotator cuff (RC Group), Achilles tendon (AT Group), or patellar tendon (PT Group) treated with PRP.

### 2.2. Inclusion and Exclusion Criteria

Participants were patients of both sexes over 18 years old, with chronic pain lasting more than 3 months, diagnosed with mid-portion tendinopathy in the supraspinatus tendon of the rotator cuff, in the Achilles tendon, or in the patellar tendon. These patients were treated with a combination of intratendinous and peritendinous PRP injections at the target tendon. The diagnostic procedures were conducted by orthopedic surgeons using magnetic resonance imaging.

The exclusion criteria were: patients contraindicated for PRP treatment due to comorbidities (such as infections, malignancies or hematologic disorders); associated joint pathologies including acute tendon injuries, bone marrow lesions, tendon ruptures and osteoarthritis; previous treatments in the 6 months prior to PRP treatment, PRP infiltrations following or complementary to a surgical procedure, patients who did not complete the treatment application protocol; new joint injuries or interventions that were unrelated to tendon pathology; and lack of follow-up after treatment.

### 2.3. Platelet-Rich Plasma Preparation

A total of 48 mL of venous blood was drawn into 9-mL tubes containing 3.8% (*w*/*v*) sodium citrate and then centrifuged at 580× *g* for 8 min at room temperature (BTI Biotechnology Institute, Vitoria-Gasteiz, Spain). The 2-mL layer of plasma situated above the red blood cell fraction—excluding the buffy coat—was collected. This portion of plasma had a moderate platelet concentration (approximately twice that of peripheral blood) and lacked erythrocytes and leukocytes, constituting leukocyte-poor PRP. Calcium chloride (10% *w*/*v*) was added as an activating agent immediately before each injection. All steps were carried out under sterile conditions, and the preparation and application process took a total of 20 min.

### 2.4. Platelet-Rich Plasma Quality Control

Blood and PRP samples were collected and analyzed in the Sysmex XS-1000i hematology analyzer (Sysmex, Kobe, Japan) to verify that the resulting PRP elaborated complies with the parameters indicated by the manufacturer. These assays included the analysis of the different cell populations (red blood cells, white blood cells and platelets).

The mean PRP platelet concentration was (371.9 ± 87.1) × 10^3^ platelets/μL, reaching a concentration factor of 2.0 ± 0.3, with no leukocytes or erythrocytes. In accordance with the latest universal coding system (UCS) and minimum reporting requirements for PRP studies, the PRP used in this study was 13-00-11 [20]. The code is a sequence of 6 digits grouped into pairs indicating the parameters of platelet composition, purity and activation with the aim of unifying the way PRP is classified for comparison. The characteristics of the PRP are reported in Table 1.

### 2.5. Platelet-Rich Plasma Application Protocol

**Figure 1:**
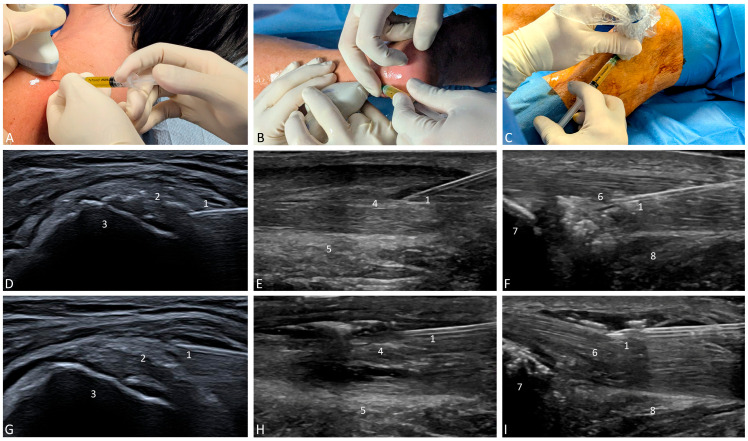
Application of PRP in tendinopathies. PRP was administered into the supraspinatus tendon of the rotator cuff (**A**,**D**,**G**), Achilles tendon (**B**,**E**,**H**), and patellar tendon (**C**,**F**,**I**). The injections were ultrasound-guided (**A**–**C**), using both an intratendinous (**D**–**F**) and peritendinous (**G**–**I**) approach. When administering PRP, a hyperechoic signal was observed. 1: needle; 2: supraspinatus tendon; 3: greater tuberosity; 4: Achilles tendon; 5: Kager’s fat pad; 6: patellar tendon; 7: patella; 8: Hoffa’s fat pad.

The PRP application followed the same guidelines for the three locations of tendons treated (supraspinatus, Achilles, patellar) (Figure 1). The injections were ultrasound-guided and performed by an orthopedic surgeon with the assistance of a radiologist to ensure accurate administration. The PRP application protocol included three PRP administrations at intervals of two weeks.

Once the patient has been positioned for optimal access to the affected tendon, the PRP was activated with CaCl_2_ and applied via both intratendinous and peritendinous routes. Small diameter syringes were used to increase the infiltration pressure, facilitating PRP to diffuse throughout the tendon tissue.

The needle was positioned parallel to the ultrasound probe, with the transducer aligned in the long-axis relative to the tendon, allowing near-parallel insertion for targeted PRP application along the tendon fibers.

Initially, the needle was carefully inserted to reach the fascicular perineurium, enabling intratendinous infiltration. The infiltration was performed not only in the area of the lesion but also in the adjacent areas to stimulate the cells of the healthy tissue and their paracrine action. Afterward, the needle was slowly positioned just above or below the tendon to perform the peritendinous injection, surrounding the tendon. During peritendon infiltration, gentle separation of adjacent tissues resulted in a hydrodissection effect. Between 8 and 12 mL were injected in total, depending on the size of the tendon using a 21-gauge needle. After injection, patients may experience soreness for 2–3 days, in which case we recommended relative rest and local ice. Patients were advised to avoid taking nonsteroidal anti-inflammatory drugs (NSAIDs), but analgesics and loading were allowed depending on pain intensity.

### 2.6. Outcome Measures

Patients in the RC group filled out the Disabilities of the Arm, Shoulder, and Hand (DASH) scale, patients in the AT group completed the Victorian Institute of Sport Assessment—Achilles (VISA-A) questionnaire, and patients in the PT group filled out the Victorian Institute of Sport Assessment—Patella (VISA-P) questionnaire. The results of the DASH questionnaire were transformed (100 minus the score obtained) so that, as with the VISA-A and Visa-P questionnaires, 0 points was the worst outcome and 100 was the best, thus enabling comparison of the data from the three groups. The scales were completed before treatment (baseline) and after 6 months of follow-up. Concomitant analgesic/anti-inflammatory medication was prohibited 48 h prior to assessment.

The primary efficacy criterion was a change from the baseline score. Success rates were calculated according to the minimal clinically important improvement (MCII), calculated as an improvement in the score from baseline of at least 11 points in DASH scale for the RC Group [21], 14 points in the VISA-A for the AT Group [22], and 13 points in the VISA-P for the PT Group [23]. Data analyses were performed by blinded investigators.

All complications and adverse events were assessed and reported during patient visits.

### 2.7. Statistical Analysis

Demographic and clinical variables were summarized as mean and standard deviation. The Gaussian distribution of the samples was determined using the Shapiro–Wilk test. Within-group (pre-post) comparisons for each tendon group were performed using paired Student’s t-tests for continuous variables. Between-group comparisons of changes from baseline were conducted using one-way ANOVA. Homogeneity of variance was assessed using Levene’s test. Post hoc pairwise comparisons were adjusted for multiple testing using the Bonferroni correction. Categorical variables were analyzed using the chi-squared test. Chi-squared tests were conducted for categorical data. Pairwise comparisons were quantified with Hedges’ g to estimate effect size; effect sizes were interpreted as small (g = 0.2), moderate (g = 0.5), or large (g = 0.8). A multivariate logistic regression model was constructed with MCII achievement (no/yes) as the dependent variable and sex (men/women), age, and tendon location (three-level categorical variable) as independent predictors. Multicollinearity among predictors was evaluated using variance inflation factors (VIF) and model fit was assessed using the Hosmer–Lemeshow goodness-of-fit test. Data were considered statistically significant when *p* < 0.05. Statistical analysis was performed with SPSS 20.0 (SPSS, Chicago, IL, USA).

## 3. Results

### 3.1. Demographics and Patient Characteristics

**Figure 2:**
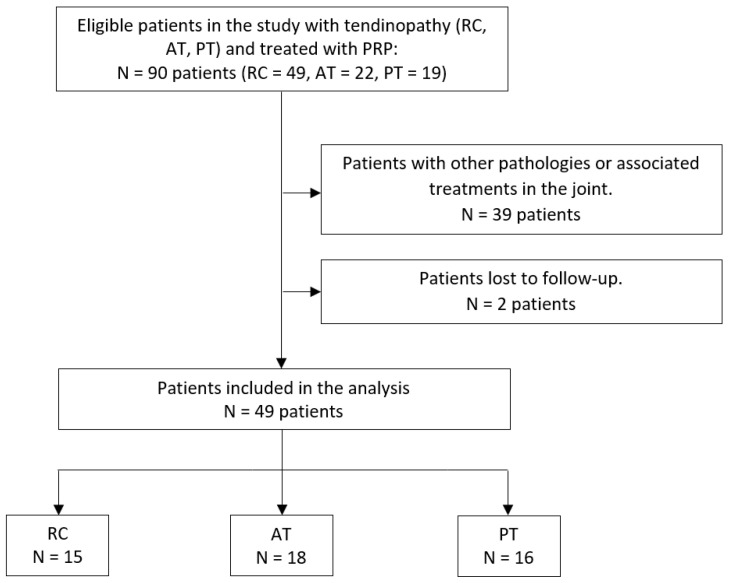
Study flowchart. RC: rotator cuff; AT: Achilles tendon; PT: patellar tendon; PRP: Platelet-Rich Plasma.

A total of 49 patients were included in the final analysis (Figure 2). The mean age was 51.4 ± 11.7 years and the number of women was 13 (26.5%). Table 2 shows the characteristics of patients classified into different groups. The mean age of the PT Group was significantly lower than in the other groups (*p* < 0.001).

### 3.2. Hematological Blood and PRP Characteristics Across Groups

There were no significant differences in any hematological blood or PRP values between the three groups (*p* > 0.05; Table 3).

### 3.3. Effectiveness of PRP in Tendon Symptomatology

The overall number of responders at 6 months of treatment was 33 (67.4%). There was a significant improvement between the baseline score (45.4 ± 24.3) and the 6-month score (69.8 ± 25.5), with an average increase of 24.3 ± 27.2 points.

**Figure 3:**
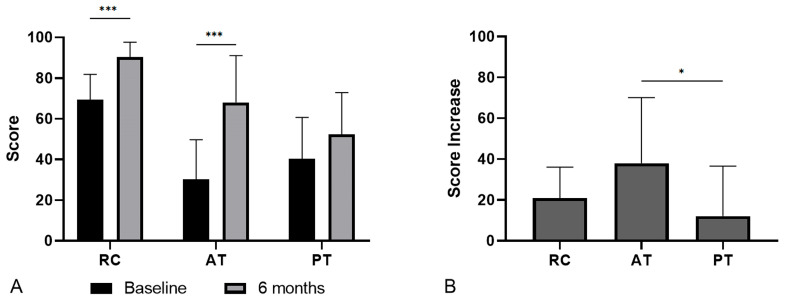
Clinical scores. Results of the scales before and after treatment (**A**) and increase in score (**B**). The RC group was evaluated using the DASH scale, the AT group using the VISA-A scale, and the PT group using the VISA-P scale (0: worst result–100: best result). RC: rotator cuff; AT: Achilles tendon; PT: patellar tendon. * *p* < 0.05; *** *p* < 0.001.

Table 4 shows the clinical scores for the different groups. Patients in the RC and AT groups experienced a significant improvement according to their scores (*p* < 0.001), which was not seen in the PT group (*p* = 0.065) (Figure 3A). In addition, the RC group had the highest values at both baseline and 6 months (*p* < 0.001), while the AT group achieved the highest improvement (*p* = 0.015) (Figure 3B).

No adverse effects related to the treatment were reported apart from pain caused by the injection, which caused discomfort for up to 24–48 h post-injection.

### 3.4. Influence of Patient Variables on PRP Response

While age was not a factor influencing response, the percentage of responders was higher in the group of women (12/13, 92.3%) than in the group of men (21/36, 58.3%) (*p* = 0.025). More specifically, this was observed in the PT Group, with 100% of women responding (4/4) compared to 33.3% of men (4/12) (*p* = 0.021).

Multivariate analysis confirmed these results, showing that female sex was a factor favoring positive responses to PRP, while tendinopathy in the patellar tendon was a negative factor. All 49 patients had complete data and were included in the model. The model showed adequate fit (Hosmer–Lemeshow *p* = 0.073) and no significant multicollinearity. The events-per-variable ratio was 11 (33 events for three predictors), meeting the recommended threshold for model stability (Table 5). Although sex and tendon location were associated with MCII achievement in this model, these findings should be interpreted with caution due to the limited sample size and the observational nature of the study.

## 4. Discussion

The main finding of this study is the clinical improvement observed after PRP treatment in rotator cuff tendinopathies (supraspinatus tendon) and Achilles tendon tendinopathies, with reduced efficacy in the patellar tendon and a better response in female patients. Regarding rotator cuff tendinopathy, these results support previous evidence that leukocyte-poor PRP (LP-PRP) may play a beneficial role. In a randomized clinical trial, the use of LP-PRP in rotator cuff repair was shown to reduce the rate of re-tears [24]. Although the present study did not involve surgical repair, biological infiltration itself appears to promote the tendon’s own healing processes. Furthermore, recent systematic reviews and meta-analysis also revealed a reduction in pain following PRP treatment, although there was heterogeneity in the studies due to differences in PRP preparation, concentration, and application protocol [25,26,27]. These findings reinforce the hypothesis that intratendinous and peritendinous infiltrations may be facilitating a reparative microenvironment that not only modulates inflammation but also improves tendon structural integrity.

Regarding the application of PRP in Achilles tendinopathy, the present study found patients in this group showed the greatest improvement. However, clinical evidence in this area remains highly variable. A recent meta-analysis of randomized clinical trials concluded that PRP does not significantly improve function or pain compared to placebo for chronic Achilles tendinopathy, and suggesting to possible risk of publication bias [28]. Furthermore, another recent meta-analysis included five controlled trials and reported that, although the overall long-term differences were inconclusive, better scores and greater pain reduction were observed at early time points [29]. The limited response reported in the literature on the use of PRP in this type of pathology could be linked to the application protocol. Usually, small volumes of PRP (around 4 mL) are administered as a single injection [30,31,32,33,34]. In contrast, the present study, administered three rounds of approximately 10 mL, using a multitarget approach combining intratendinous and peritendinous injections each time. These injections covered not only the injured area but also acted on healthy tissue, stimulating the paracrine action of these cells and enhancing their effect on tissue repair. In addition, several of the aforementioned studies use leukocyte-rich PRP (LR-PRP) [30,31,33], whereas we used LP-PRP. Although in vivo studies suggest that the application of LR-PRP may be more beneficial in the early stages of tendinopathy [35], in models of chronic tendinopathy, which are more applicable to the present study, LP-PRP showed better results and improved tendon healing [36]. However, the application of PRP —with or without leukocytes— in tendon pathology requires further research, and both types of PRP may be beneficial for the appropriate indication. Finally, it should be noted that patients in this group presented the worst initial symptoms, meaning that the potential for improvement was greater than that of patients in the other two groups.

With regard to patellar tendinopathy, the efficacy of PRP was lower than in the RC and AT groups. As discussed above, comparison with the literature is challenging due to contradictory data arising from heterogeneity in PRP type and protocols [37]. Moreover, most studies employ protocols that differ from those used in the present work, with smaller volumes and single injections, leading to varied results [37,38,39,40]. It should be noted that the group of patients with patellar tendinopathy was significantly younger than the patients in the other two groups, which may be due to its higher prevalence among the physically active population [41]. Both the athletic demands and the expectations for recovery and return to activity may be higher in this population [42,43,44], making it more difficult to reach the functional requirements, potentially resulting in poorer final outcomes.

The lower response observed in patellar tendinopathy compared to that of the rotator cuff and Achilles tendon could also be due to the anatomical, biomechanical, and biological differences inherent to this tissue. The patellar tendon, which acts as a “ligamentous tendon”, is subjected to extremely high tensile and compressive loads during jumping, landing, and deceleration. This mechanical stress is combined with repeated compression against the lower pole of the patella during flexion, an area characterized by poor vascularization. All of this creates a mechanically challenging microenvironment that induces degenerative structural changes that lead to fibrillar degeneration and limited repair [45,46,47]. Intraosseous injections into the patella [48] could improve the effectiveness of patellar tendinopathy treatment, given the particular importance of the bone insertion of this tendon. The direct application of PRP to bone and subchondral tissue has been shown to stimulate cell populations involved in tissue repair [49] and improve the effectiveness of this treatment in conditions such as osteoarthritis [50]. Targeting on the bone insertion of the tendon would broaden the range of action of PRP by stimulating a greater number of cell populations that would participate in tissue healing. Indeed, bone-tendon communication is crucial in maintaining this structure, with high mechanobiological signaling and key cell populations in its pathophysiology [51,52]. Further research in this area is needed to understand the biological mechanisms and to improve application protocols.

Finally, it is noteworthy that women exhibited a higher response rate, especially in the patellar tendon. This is consistent with results from a previous retrospective study, in which female sex was associated with a higher probability of significant improvement at six months after PRP treatment for tendinopathy [53]. As with other pathologies [54,55,56], these differences may be due to sociocultural, mechanical, anatomical, or biological causes. For instance, Hansen et al. [57] observed that estrogens modulated collagen synthesis, fibrillar organization, and tenocyte activity, potentially leading to a more stable extracellular matrix and greater repair efficiency in women. However, further research is needed in this area, and future studies should stratify patients to assess whether the effectiveness of PRP varies according to sex and whether treatment should be adapted [58].

The main limitations of this study are the small sample size and the lack of a control group. Although our results suggest higher response rates in women, the small number of female participants makes these findings exploratory. Therefore, conclusions regarding sex differences should be interpreted with caution and require confirmation in larger studies. In addition, we did not perform a detailed analysis of factors such as mechanical load or the degree of tendon degeneration using imaging, which could help to better explain the results obtained. Similarly, we did not have follow-up data beyond six months, which limits our understanding of the durability of clinical improvement. Another limitation was that different patient-reported outcome measures were used for each group, which limits direct comparability; however, this approach was necessary to capture the specific functional impact of each distinct tendinopathy, and some comparability can still be inferred through normalized scoring across the scales, partially mitigating this concern. Finally, studies on the molecular composition of PRP, in addition to its cellular components, could shed more light on the responsiveness of patients to PRP.

The results of this study are clinically relevant, as they suggest that repeated injections of large volumes of multi-target PRP are safe and produce clinical improvement in the treatment of tendinopathies. This efficacy was influenced by patient variables such as location of tendinopathy or sex.

## 5. Conclusions

Repeated intratendinous and peritendinous PRP injections in rotator cuff tendinopathy, Achilles tendon, and patellar tendon improved joint function 6 months after treatment. This improvement was less pronounced in patients with patellar tendinopathy and the proportion of responders was higher among women.

## Figures and Tables

**Table 1:** Characteristics of Platelet Rich-Plasma.

**1. PRP Preparation**	
Initial blood volume	48 mL
Anticoagulant	Sodium citrate 3.8% (*w*/*v*)
System	Close
Centrifugation	Yes
* number*	*1*
* speed*	*580 g/8 min*
Final PRP volume	12 mL
**2. PRP Characteristics**	
PRP Type	13-00-11
MPV	10.3 ± 0.8 fL
Red Blood Cells	<0.1 × 10^6^/μL
White Blood Cells	<0.1 × 10^6^/μL
Activation	CaCl_2_ (10% *w*/*v*)
**3. Application Characteristics**	
Formulation type	Liquid
Administration route	intratendinous and peritendinous
Dosage	3 injections with a two-week interval
Volume	8–12 mL
Dose (range of platelets)	2.3 × 10^9^–5.5 × 10^9^
Tissue	Tendon (supraspinatus, Achilles, patella)
Pathology	Tendinopathy

PRP: Platelet-Rich Plasma; MPV: mean platelet volume.

**Table 2:** Demographic and clinical characteristics.

	RC Group	AT Group	PT Group	*p* Value
N	15	18	16	
Age, mean ± SD	55.7 ± 7.0	57.4 ± 11.3	40.6 ± 7.6 ^a^	<0.001
Female, *n* (%)	6 (40.0%)	3 (16.7%)	4 (25.0%)	0.314

RC: rotator cuff; AT: Achilles tendon; PT: patellar tendon. SD: standard deviation; ^a^ significant difference compared to the other groups.

**Table 3:** Hematological values of blood and PRP.

	RC Group	AT Group	PT Group	*p* Value
WBC Blood (×10^3^/µL), mean ± SD	7.3 ± 3.0	5.1 ± 1.2	5.9 ± 2.2	0.182
PLT Blood (×10^3^/µL), mean ± SD	203.8 ± 57.7	199.2 ± 45.9	178.4 ± 45.8	0.624
MPV Blood (fL), mean ± SD	10.3 ± 0.6	10.0 ± 0.6	10.1 ± 0.8	0.235
WBC PRP (×10^3^/µL), mean ± SD	0.1 ± 0.1	0.1 ± 0.1	0.1 ± 0.1	0.868
PLT PRP (×10^3^/µL), mean ± SD	396.8 ± 81.8	409.6 ± 90.7	336.1 ± 79.5	0.167
MPV PRP (fL), mean ± SD	9.7 ± 0.5	4.4 ± 0.6	9.3 ± 1.0	0.132
CF, mean ± SD	2.0 ± 0.3	2.1 ± 0.1	1.9 ± 0.4	0.568

RC: rotator cuff; AT: Achilles tendon; PT: patellar tendon; SD: standard deviation; WBC: white blood cells, PLT: platelets; MPV: mean platelet volume; PRP: Platelet-Rich Plasma; CF: concentration factor.

**Table 4:** Baseline and 6-month clinical scores.

	RC Group	AT Group	PT Group	*p* Value ^1^
Baseline, mean ± SD	69.4 ± 12.5 ^a^	30.1 ± 19.6	40.3 ± 20.3	<0.001
6 months, mean ± SD	90.4 ± 7.2 ^a^	68.3 ± 23.0	52.4 ± 38.4	<0.001
*p* value ^2^	<0.001	<0.001	0.065	
δ, mean ± SD	20.9 ± 15.1	37.9 ± 32.1 ^b^	12.1 ± 24.4	0.015
Effect size (g)	0.63 (RC vs. AT)	0.86 (AT vs. PT)	0.41 (RC vs. PT)	
MCII, n (%)	11 (73.3)	14 (75.0)	8 (50.0)	0.190

RC: rotator cuff tendinosis; AT: Achilles tendon; PT: patellar tendon; SD: standard deviation; MCII: minimal clinically important improvement. ^1^ Comparison between groups; ^2^ comparison between timepoints; ^a^ significant difference compared to the other groups; ^b^ significant difference compared to PT group.

**Table 5:** Multivariate regression analysis for response at 6 months.

Variable	B	*p* Value	95% CI	OR	VIF	Tolerance
Tendon (1)	1.517	0.148	0.584 to 35.587	4.561	1.407	0.711
Tendon (2)	2.382	0.047	1.033 to 113.489	10.826		
Age	−0.044	0.295	0.882 to 1.039	0.957	1.384	0.722
Sex (3)	−2.450	0.032	0.009 to 0.812	0.086	1.020	0.981

B: coefficient, CI: confidence interval; OR: Odds ratio; VIF: variance inflation factors (1): RC Group vs. PT Group (reference); (2): AT Group vs. PT Group (reference); (3): male vs. female (reference).

## Data Availability

The original contributions presented in this study are included in the article. Further inquiries can be directed to the corresponding author.

## Abbreviations

The following abbreviations are used in this manuscript:

AT	Achilles Tendon
DASH	Disabilities of the Arm, Shoulder, and Hand
MCII	Minimal Clinically Important Improvement
PRP	Platelet-Rich Plasma
PT	Patellar Tendon
RC	Rotator Cuff
VISA-A	Victorian Institute of Sport Assessment—Achilles
VISA-P	Victorian Institute of Sport Assessment—Patella

## References

[1] Zhou Y., Wang J.H.-C. (2016). PRP Treatment Efficacy for Tendinopathy: A Review of Basic Science Studies. Biomed. Res. Int..

[2] Thomopoulos S., Parks W.C., Rifkin D.B., Derwin K.A. (2015). Mechanisms of Tendon Injury and Repair. J. Orthop. Res..

[3] Millar N.L., Silbernagel K.G., Thorborg K., Kirwan P.D., Galatz L.M., Abrams G.D., Murrell G.A.C., McInnes I.B., Rodeo S.A. (2021). Tendinopathy. Nat. Rev. Dis. Primers.

[4] Aicale R., Tarantino D., Maffulli N. (2018). Surgery in Tendinopathies. Sports Med. Arthrosc. Rev..

[5] Irby A., Gutierrez J., Chamberlin C., Thomas S.J., Rosen A.B. (2020). Clinical Management of Tendinopathy: A Systematic Review of Systematic Reviews Evaluating the Effectiveness of Tendinopathy Treatments. Scand. J. Med. Sci. Sports.

[6] Cooper K., Alexander L., Brandie D., Brown V.T., Greig L., Harrison I., MacLean C., Mitchell L., Morrissey D., Moss R.A. (2023). Exercise Therapy for Tendinopathy: A Mixed-Methods Evidence Synthesis Exploring Feasibility, Acceptability and Effectiveness. Health Technol. Assess..

[7] Puzzitiello R.N., Patel B.H., Forlenza E.M., Nwachukwu B.U., Allen A.A., Forsythe B., Salzler M.J. (2020). Adverse Impact of Corticosteroids on Rotator Cuff Tendon Health and Repair: A Systematic Review of Basic Science Studies. Arthrosc. Sports Med. Rehabil..

[8] Eliasberg C.D., Rodeo S.A. (2025). Orthobiologics for Tendon Injuries. Clin. Sports Med..

[9] Wu W.-S., Chen L.-R., Chen K.-H. (2025). Platelet-Rich Plasma (PRP): Molecular Mechanisms, Actions and Clinical Applications in Human Body. Int. J. Mol. Sci..

[10] Del Bue M., Riccò S., Conti V., Merli E., Ramoni R., Grolli S. (2007). Platelet Lysate Promotes in Vitro Proliferation of Equine Mesenchymal Stem Cells and Tenocytes. Vet. Res. Commun..

[11] Wang X., Qiu Y., Triffitt J., Carr A., Xia Z., Sabokbar A. (2012). Proliferation and Differentiation of Human Tenocytes in Response to Platelet Rich Plasma: An In Vitro and In Vivo Study. J. Orthop. Res..

[12] Rui Y.-F., Lui P.P.Y., Li G., Fu S.C., Lee Y.W., Chan K.M. (2010). Isolation and Characterization of Multipotent Rat Tendon-Derived Stem Cells. Tissue Eng. Part A.

[13] Bendinelli P., Matteucci E., Dogliotti G., Corsi M.M., Banfi G., Maroni P., Desiderio M.A. (2010). Molecular Basis of Anti-Inflammatory Action of Platelet-Rich Plasma on Human Chondrocytes: Mechanisms of NF-ΚB Inhibition via HGF. J. Cell. Physiol..

[14] Ling S.K.-K., Mak C.T.-K., Lo J.P.-Y., Yung P.S.-H. (2024). Effect of Platelet-Rich Plasma Injection on the Treatment of Achilles Tendinopathy: A Systematic Review and Meta-Analysis. Orthop. J. Sports Med..

[15] Bahadir B., Sarikaya B. (2024). Platelet-Rich Plasma in the Management of Rotator Cuff Tendinopathy. Jt. Dis. Relat. Surg..

[16] Wang S., Lyu B. (2025). Effectiveness of Injection Strategies on Patients With Patellar Tendonitis (Jumpers’ Knee): A Network Meta-Analysis of Randomized Controlled Trials. Sports Health.

[17] Chalidis B., Givissis P., Papadopoulos P., Pitsilos C. (2023). Molecular and Biologic Effects of Platelet-Rich Plasma (PRP) in Ligament and Tendon Healing and Regeneration: A Systematic Review. Int. J. Mol. Sci..

[18] Sánchez M., Jorquera C., de Dicastillo L.L., Fiz N., Knörr J., Beitia M., Aizpurua B., Azofra J., Delgado D. (2022). Real-World Evidence to Assess the Effectiveness of Platelet-Rich Plasma in the Treatment of Knee Degenerative Pathology: A Prospective Observational Study. Ther. Adv. Musculoskelet. Dis..

[19] Filardo G., Di Matteo B., Kon E., Merli G., Marcacci M. (2018). Platelet-Rich Plasma in Tendon-Related Disorders: Results and Indications. Knee Surg. Sports Traumatol. Arthrosc..

[20] Kon E., Di Matteo B., Delgado D., Cole B.J., Dorotei A., Dragoo J.L., Filardo G., Fortier L.A., Giuffrida A., Jo C.H. (2020). Platelet-Rich Plasma for the Treatment of Knee Osteoarthritis: An Expert Opinion and Proposal for a Novel Classification and Coding System. Expert Opin. Biol. Ther..

[21] Galardini L., Coppari A., Pellicciari L., Ugolini A., Piscitelli D., La Porta F., Bravini E., Vercelli S. (2024). Minimal Clinically Important Difference of the Disabilities of the Arm, Shoulder and Hand (DASH) and the Shortened Version of the DASH (QuickDASH) in People With Musculoskeletal Disorders: A Systematic Review and Meta-Analysis. Phys. Ther..

[22] Lagas I.F., van der Vlist A.C., van Oosterom R.F., van Veldhoven P.L.J., Reijman M., Verhaar J.A.N., de Vos R.-J. (2021). Victorian Institute of Sport Assessment-Achilles (VISA-A) Questionnaire-Minimal Clinically Important Difference for Active People With Midportion Achilles Tendinopathy: A Prospective Cohort Study. J. Orthop. Sports Phys. Ther..

[23] Khoury M.A., Chamari K., Tabben M., Alkhelaifi K., Ricardo T., Damián C., D’hooghe P. (2021). Expanded Adipose Derived Mesenchymal Stromal Cells Are Effective in Treating Chronic Insertional Patellar Tendinopathy: Clinical and MRI Evaluations of a Pilot Study. J. Exp. Orthop..

[24] Rossi L.A., Gorodischer T.D., Camino P., Brandariz R.N., Tanoira I., Piuzzi N.S., Ranalletta M. (2024). Leukocyte-Poor Platelet-Rich Plasma as an Adjuvant to Arthroscopic Rotator Cuff Repair Reduces the Retear Rate But Does Not Improve Functional Outcomes: A Double-Blind Randomized Controlled Trial. Am. J. Sports Med..

[25] Lin M.-T., Wei K.-C., Wu C.-H. (2020). Effectiveness of Platelet-Rich Plasma Injection in Rotator Cuff Tendinopathy: A Systematic Review and Meta-Analysis of Randomized Controlled Trials. Diagnostics.

[26] Chen X., Jones I.A., Park C., Vangsness C.T. (2018). The Efficacy of Platelet-Rich Plasma on Tendon and Ligament Healing: A Systematic Review and Meta-Analysis with Bias Assessment. Am. J. Sports Med..

[27] Roy M., Reddy M.H., Das D., Priyanshu, Chandrakar D., Elavarasu A.M. (2025). Effectiveness of Platelet-Rich Plasma in Treating Rotator Cuff Tendinopathy: A Systematic Review and Meta-Analysis. J. Orthop. Case Rep..

[28] Barreto E.S.R., Antunes Júnior C.R., Silva I.C., Alencar V.B., Faleiro T.B., Kraychete D.C. (2025). Is Platelet-Rich Plasma Effective in Treating Achilles Tendinopathy? A Meta-Analysis of Randomized Clinical Trials. Clin. Orthop. Relat. Res..

[29] Desouza C., Dubey R., Shetty V. (2023). Platelet-Rich Plasma in Chronic Achilles Tendinopathy. Eur. J. Orthop. Surg. Traumatol..

[30] de Jonge S., de Vos R.J., Weir A., van Schie H.T.M., Bierma-Zeinstra S.M.A., Verhaar J.A.N., Weinans H., Tol J.L. (2011). One-Year Follow-up of Platelet-Rich Plasma Treatment in Chronic Achilles Tendinopathy: A Double-Blind Randomized Placebo-Controlled Trial. Am. J. Sports Med..

[31] de Vos R.J., Weir A., van Schie H.T.M., Bierma-Zeinstra S.M.A., Verhaar J.A.N., Weinans H., Tol J.L. (2010). Platelet-Rich Plasma Injection for Chronic Achilles Tendinopathy: A Randomized Controlled Trial. JAMA.

[32] Boesen A.P., Boesen M.I., Hansen R., Barfod K.W., Lenskjold A., Malliaras P., Langberg H. (2020). Effect of Platelet-Rich Plasma on Nonsurgically Treated Acute Achilles Tendon Ruptures: A Randomized, Double-Blinded Prospective Study. Am. J. Sports Med..

[33] Krogh T.P., Ellingsen T., Christensen R., Jensen P., Fredberg U. (2016). Ultrasound-Guided Injection Therapy of Achilles Tendinopathy With Platelet-Rich Plasma or Saline: A Randomized, Blinded, Placebo-Controlled Trial. Am. J. Sports Med..

[34] Kearney R.S., Parsons N., Costa M.L. (2013). Achilles Tendinopathy Management: A Pilot Randomised Controlled Trial Comparing Platelet-Richplasma Injection with an Eccentric Loading Programme. Bone Jt. Res..

[35] Jiang G., Wu Y., Meng J., Wu F., Li S., Lin M., Gao X., Hong J., Chen W., Yan S. (2020). Comparison of Leukocyte-Rich Platelet-Rich Plasma and Leukocyte-Poor Platelet-Rich Plasma on Achilles Tendinopathy at an Early Stage in a Rabbit Model. Am. J. Sports Med..

[36] Yan R., Gu Y., Ran J., Hu Y., Zheng Z., Zeng M., Heng B.C., Chen X., Yin Z., Chen W. (2017). Intratendon Delivery of Leukocyte-Poor Platelet-Rich Plasma Improves Healing Compared With Leukocyte-Rich Platelet-Rich Plasma in a Rabbit Achilles Tendinopathy Model. Am. J. Sports Med..

[37] Scott A., LaPrade R.F., Harmon K.G., Filardo G., Kon E., Della Villa S., Bahr R., Moksnes H., Torgalsen T., Lee J. (2019). Platelet-Rich Plasma for Patellar Tendinopathy: A Randomized Controlled Trial of Leukocyte-Rich PRP or Leukocyte-Poor PRP Versus Saline. Am. J. Sports Med..

[38] Di Matteo B., Filardo G., Kon E., Marcacci M. (2015). Platelet-Rich Plasma: Evidence for the Treatment of Patellar and Achilles Tendinopathy—A Systematic Review. Musculoskelet. Surg..

[39] van der Heijden R.A., Stewart Z., Moskwa R., Liu F., Wilson J., Hetzel S.J., Thelen D., Heiderscheit B., Kijowski R., Lee K. (2024). Platelet-Rich Plasma for Patellar Tendinopathy: A Randomized Controlled Trial Correlating Clinical Outcomes and Quantitative Imaging. Radiol. Adv..

[40] Herrero C., Wasterlain A., Bloom D.A., Pham H., Weinberg M., Dragoo J.L., Strauss E.J. (2024). Leukocyte-Poor Platelet-Rich Plasma as a Treatment for Patellar Tendinopathy A Multicenter, Randomized Controlled Trial. Bull. Hosp. Jt. Dis..

[41] Nutarelli S., da Lodi C.M.T., Cook J.L., Deabate L., Filardo G. (2023). Epidemiology of Patellar Tendinopathy in Athletes and the General Population: A Systematic Review and Meta-Analysis. Orthop. J. Sports Med..

[42] Webster K.E., Nagelli C.V., Hewett T.E., Feller J.A. (2018). Factors Associated With Psychological Readiness to Return to Sport After Anterior Cruciate Ligament Reconstruction Surgery. Am. J. Sports Med..

[43] Pereira G.F., McLean S.A., Tkacik T.J., Swor R.A., Jones J.S., Lee D.C., Peak D.A., Domeier R.M., Rathlev N.K., Hendry P.L. (2014). Pain, Distress, and Anticipated Recovery for Older versus Younger Emergency Department Patients after Motor Vehicle Collision. BMC Emerg. Med..

[44] Montemurro L., Carrière J.S., Durand M., Coutu M. (2025). Physical Therapists’ Expectations Regarding the Recovery of Work-Disabled Individuals Aged 55 and Over. Musculoskelet. Care.

[45] Basso O., Johnson D.P., Amis A.A. (2001). The Anatomy of the Patellar Tendon. Knee Surg. Sports Traumatol. Arthrosc..

[46] Toumi H., Higashiyama I., Suzuki D., Kumai T., Bydder G., McGonagle D., Emery P., Fairclough J., Benjamin M. (2006). Regional Variations in Human Patellar Trabecular Architecture and the Structure of the Proximal Patellar Tendon Enthesis. J. Anat..

[47] Peniche Silva C.J., Müller S.A., Quirk N., De la Vega R.E., Coenen M.J., Evans C.H., Balmayor E.R., van Griensven M. (2022). Enthesis: Not the Same in Each Localisation—A Molecular, Histological and Biomechanical Study. Eur. Cell Mater..

[48] Sánchez M., Fiz N., Guadilla J., Padilla S., Anitua E., Sánchez P., Delgado D. (2014). Intraosseous Infiltration of Platelet-Rich Plasma for Severe Knee Osteoarthritis. Arthrosc. Tech..

[49] Ganguly P., Fiz N., Beitia M., Owston H.E., Delgado D., Jones E., Sánchez M. (2022). Effect of Combined Intraosseous and Intraarticular Infiltrations of Autologous Platelet-Rich Plasma on Subchondral Bone Marrow Mesenchymal Stromal Cells from Patients with Hip Osteoarthritis. J. Clin. Med..

[50] Sánchez M., Delgado D., Pompei O., Pérez J.C., Sánchez P., Garate A., Bilbao A.M., Fiz N., Padilla S. (2019). Treating Severe Knee Osteoarthritis with Combination of Intra-Osseous and Intra-Articular Infiltrations of Platelet-Rich Plasma: An Observational Study. Cartilage.

[51] Xiao H., Wen B., Yan D., Li Q., Yang Y., Yin X., Chen D., Liu J. (2023). Hot Spots and Frontiers in Bone-Tendon Interface Research: A Bibliometric Analysis and Visualization from 2000 to 2023. Front. Surg..

[52] Chen Z., Jin M., He H., Dong J., Li J., Nie J., Wang Z., Xu J., Wu F. (2023). Mesenchymal Stem Cells and Macrophages and Their Interactions in Tendon-Bone Healing. J. Orthop. Transl..

[53] Unlu M.C., Kivrak A., Kayaalp M.E., Birsel O., Akgun I. (2017). Peritendinous Injection of Platelet-Rich Plasma to Treat Tendinopathy: A Retrospective Review. Acta Orthop. Traumatol. Turc..

[54] Sánchez M., Jorquera C., López de Dicastillo L., Martínez N., Espregueira-Mendes J., Vergés J., Azofra J., Delgado D. (2024). Women Show a Positive Response to Platelet-Rich Plasma despite Presenting More Painful Knee Osteoarthritis than Men. Knee Surg. Sports Traumatol. Arthrosc..

[55] Hopper H., Adsit M., Reiter C.R., Satalich J.R., Schmidt R.C., Peri M.I., Cyrus J.W., Vap A.R. (2024). Female Sex, Older Age, Earlier Surgery, Anticoagulant Use, and Meniscal Repair Are Associated With Increased Risk of Manipulation Under Anesthesia or Lysis of Adhesions for Arthrofibrosis After Anterior Cruciate Ligament Reconstruction: A Systematic Review. Arthroscopy.

[56] Sánchez M., Yarza I., Jorquera C., Aznar J.M., de Dicastillo L.L., Valente C., Andrade R., Espregueira-Mendes J., Celorrio D., Aizpurua B. (2025). Genetics, Sex and the Use of Platelet-Rich Plasma Influence the Development of Arthrofibrosis after Anterior Cruciate Ligament Reconstruction. J. Exp. Orthop..

[57] Hansen M., Kjaer M. (2014). Influence of Sex and Estrogen on Musculotendinous Protein Turnover at Rest and after Exercise. Exerc. Sport Sci. Rev..

[58] Filardo G., de Girolamo L., Kon E., Hirschmann M.T., Karlsson J. (2024). Bridging the Gender Data-Gap in Studies of Musculoskeletal Research. Knee Surg. Sports Traumatol. Arthrosc..

